# Validation of syngeneic mouse models of melanoma and non-small cell lung cancer for investigating the anticancer effects of the soy-derived peptide Lunasin

**DOI:** 10.12688/f1000research.9661.2

**Published:** 2017-02-22

**Authors:** Bharat Devapatla, Chris Shidal, Kavitha Yaddanapudi, Keith R. Davis

**Affiliations:** 1 Department of Pharmacology and Toxicology, University of Louisville School of Medicine, , Louisville, KY, 40402, USA; 2 Department of Medicine and James Graham Brown Cancer Center, University of Louisville School of Medicine, Louisville, KY, 40402, USA; 3 Department of Biology and Biotechnology Program, Indiana University, Bloomington, IN, 47405, USA

**Keywords:** Lung cancer, Melanoma, Syngeneic tumor model, LLC, B16-F0

## Abstract

***Background***: Lunasin is a naturally occurring peptide present in soybean that has both chemopreventive and therapeutic activities that can prevent cellular transformation and inhibit the growth of several human cancer types. Recent studies indicate that Lunasin has several distinct potential modes of action including suppressing integrin signaling and epigenetic effects driven by modulation of histone acetylation. In addition to direct effects on cancer cells, Lunasin also has effects on innate immunity that may contribute to its ability to inhibit tumor growth *in vivo*.

***Methods*** *:* Standard assays for cell proliferation and colony formation were used to assess Lunasin’s *in vitro* activity against murine Lewis lung carcinoma (LLC) and B16-F0 melanoma cells.  Lunasin’s *in vivo* activity was assessed by comparing the growth of tumors initiated by subcutaneous implantation of LLC or B16-F0 cells in Lunasin-treated and untreated C57BL/6 mice.

***Results*** *:* Lunasin was found to inhibit growth of murine LLC cells and murine B16-F0 melanoma cells *in vitro* and in wild-type C57BL/6 mice.  The effects of Lunasin in these two mouse models were very similar to those previously observed in studies of human non-small cell lung cancer and melanoma cell lines.

***Conclusions*** *:* We have now validated two established syngeneic mouse models as being responsive to Lunasin treatment.  The validation of these two *in vivo* syngeneic models will allow detailed studies on the combined therapeutic and immune effects of Lunasin in a fully immunocompetent mouse model.

## Introduction

Lunasin is a multifunctional bioactive peptide present as a component of the storage protein fraction in soybean seeds and in soy-derived food products ^1– 4^. Studies from several laboratories have documented that Lunasin has both chemopreventive activity that inhibits cellular transformation by carcinogens or oncogenes ^5– 7^ and chemotherapeutic activity against multiple human cancer types ^8– 15^. Taken together, these observations suggest that Lunasin may be one of the factors responsible for the lower cancer rates observed in people who consume high-soy diets ^1– 3^. One intriguing aspect of Lunasin is that this 44 amino acid peptide has at least three potential functional domains; a polyaspartic-acid C-terminal tail that binds Lunasin to the core histones H3 and H4 ^7, 16– 18^, a tripeptide Arg-Gly-Asp (RGD) domain that can serve as a recognition signal for specific integrins ^9, 16, 19^, and a putative helical chromatin binding domain ^3, 11^. An important component of Lunasin uptake appears to be mediate by internalization via the integrin recycling pathway, with the integrin αVβ3 being a key factor ^15, 16, 19^.

Our previous studies found that native Lunasin purified from soybean has therapeutic activity against established human non-small cell lung cancer (NSCLC) and melanoma cell lines both *in vitro* and *in vivo* ^8, 15^. In the case of NSCLC, *in vitro* studies suggested that a primary mechanism of action was the inhibition of proliferation caused by inhibition of integrin signaling and decreased retinoblastoma protein phosphorylation ^15, 16, 20^. In the case of melanoma, Lunasin caused a significant decrease in putative cancer stem cells by causing these cells to switch phenotypes to a cell type expressing higher levels of the transcription factor MITF and one of its downstream targets, tyrosinase. In addition, decreased levels of the stemness protein Nanog were also observed ^8^. Our recent unpublished studies suggest that Lunasin effects on melanoma cells are also mediated, at least in part, by effects on integrin signaling ^21^. These results, along with a recent report on the effects of Lunasin on colon cancer stem-like cells ^10^, suggest the exciting possibility that Lunasin can be used to target cancer stem cells. In addition, the currently available data suggest that Lunasin does possess attributes important for clinical utility including no obvious toxicity ^8^ and being bioavailable ^22, 23^.

One of the more recent unexpected and exciting findings regarding Lunasin’s anticancer effects is that Lunasin appears to also have immunomodulatory activity ^11, 24, 25^. Interestingly, these effects correlate with epigenetic effects and do not require the RGD domain or the polyaspartic-acid tail, thus implicating the putative chromatin-binding domain as being important ^11^. Given that Lunasin has both direct therapeutic effects on cancer cells as well as the ability to affect immunity, we were prompted to determine if syngeneic mouse cancer models could be identified where both of these activities could be studied in concert so that the relative contribution of these two different effects on the potent *in vivo* activity of Lunasin could be determined. In these studies, we demonstrate that Lunasin has significant *in vitro* and *in vivo* activity in syngeneic mouse models for lung cancer and melanoma. These syngeneic models will provide the ability to pursue studies of Lunasin action in an immunocompetent host and use genetic approaches to understand how specific genetic manipulations affect Lunasin’s ability to inhibit tumor growth and metastasis.

## Methods

### Lunasin purification

Lunasin was purified from soybean white flake (Owensboro Grain Company) as previously described ^26^ by Kentucky BioProcessing (Owensboro, KY). Analysis by sodium dodecyl sulfate polyacrylamide gel electrophoresis indicated that this Lunasin preparation had >99% purity ^8^. The purified Lunasin was diluted to a concentration of 9.3 mg/ml in sterile 50 mM sodium phosphate buffer, pH 7.4 and stored at 4°C.

### Cell lines and treatments

LLC (mouse lung carcinoma) and B16-F0 (mouse melanoma) cell lines were obtained from the American Type Culture Collection (ATTC). LLC and B16-F0 cells were cultured in DMEM medium (Invitrogen). Medium was supplemented with 10% fetal bovine serum (Invitrogen), 100 IU/mL of penicillin, and 100 μg/mL of streptomycin (Invitrogen) and cells grown at 37°C in a humidified incubator containing 5% CO _2_.

### Cell growth assay

*In vitro* cell growth inhibition was measured via a tetrazolium-based [3-(4,5-dimethylthiazol-2-yl)-5-(3-carboxymethoxyphenyl)-2-(4-sulfophenyl)-2H-tetrazolium salt (MTS) assay (Promega). Briefly, 2 × 10 ^3^ cells were plated into 96-well plates and incubated overnight. The cells were treated with the indicated concentrations of Lunasin for 72 hours in 100 µL fresh medium. Every 24 hours, cell culture media was replaced with fresh culture media amended with the indicated concentrations of Lunasin. After 72 hours, 20 µL of CellTiter 96 ^®^ AQueous One reagent (Promega) was added and incubated with the cells for 1 hour. Absorbance was measured at 490 nm using a Synergy HT plate reader (Biotek). Cell growth was estimated from the absorbance readings and has been normalized to vehicle-treated control cells. Averages of three replicates per treatment were used for analysis.

### Soft agar colony-forming assays

These assays were done as previously described ^8^ except that a 24-well per plate format was used. LLC and B16-F0 cells were plated at a density of 500 and 1,000 cells/well, respectively.

### *In vivo* tumor growth studies

Six-week-old male mice (C57 BL/6) were purchased from Harlan Laboratories (Indianapolis, IN). All procedures involving mice were carried out in accordance with the international guidelines of the Association for Assessment and Accreditation of Laboratory Animals Care with the approval of the University of Louisville Institutional Animal Care and Use Committee (Protocol # 12091). Mice were maintained in the University of Louisville Health Center animal use facility and maintained by Research Resources Facilities staff using standard approved protocols. Mice were housed in polycarbonate shoebox cages (maximum 5 mice/cage) on a ventilated rack system in a temperature controlled room operating on a timed 12 hour light/dark cycle. Mice were randomly placed into groups (6–10 mice per group) and received subcutaneous injections of LLC (1 × 10 ^5^) or B16-F0 (1 × 10 ^6^) cells suspended in 100 μL of phosphate buffered saline (PBS) in the hind flank. Tumors were measured starting 10 days post-injection up to 22 days post-injection. Tumor size was measured twice weekly using digital calipers (Mitutoyo) with an accuracy of ± 0.02 mm. Tumor volume was calculated as $\frac{w^{2}\times l}{2}$ where *w =* width and *l =* length. All mice except in control group were treated with Lunasin daily starting from the day of injection. Lunasin was administered by intraperitoneal (IP) injections in 50 mM phosphate buffer at a dose of either 10 or 30 mg Lunasin/kg body weight. In some experiments, cells were pretreated with 100 μM Lunasin for 72 hours prior to injection of cells into mice. At the end of the experiments, mice were euthanized by CO _2_ asphyxiation followed by cervical dislocation.

## Results and discussion

### *In vitro* effects of Lunasin treatment

**Figure 1.**
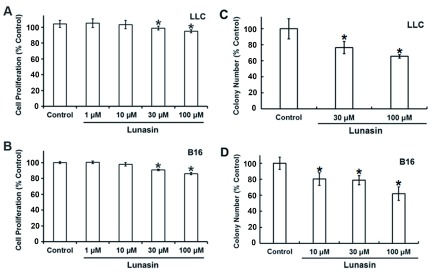
Effects of Lunasin on the *in vitro* growth of LLC and B16-F0 cells. Cells were cultured under adherent ( **A**, **B**) or non-adherent ( **C**, **D**) culture conditions and treated with the indicated concentrations of Lunasin. For adherent-culture assays, proliferation was assessed after 72 hours of treatment using a MTS assay. For non-adherent-culture assays, colonies were allowed to form over 10–18 days until colonies grew to approximately 100 μm in diameter. The number of colonies formed was counted after staining with crystal violet. Data from both assays have been normalized to the vehicle treated control and represent the mean ± S.D. An asterisk (*) indicates that a treatment was significantly different ( *p* < 0.05) from the control as determined by an unpaired student’s *t-*test.

We tested the ability of Lunasin to inhibit LLC and B16-F0 growth in both adherent and non-adherent assays. In adherent assays, Lunasin had modest dose-dependent effects on the growth of both LLC and B16-F0 cells; <10% at 30 and 100 μM Lunasin ( Figure 1A–B, Table 1). In contrast, Lunasin had substantial inhibitory activity in non-adherent colony forming assays. Both LLC and B16-F0 exhibited a dose-dependent reduction in colony formation from ^~^20% to 40% over a Lunasin concentration range of 10 to 100 μM ( Figure 1C–D, Table 2). The difference in activity observed in adherent versus non-adherent assays recapitulates our previous results using human NSCLC and melanoma cells and likely reflects differences in integrin expression profiles under these distinct culture conditions ^8, 15^. The sensitivity of the mouse cell lines were comparable to that observed for human NSCLC and melanoma cells. Growth inhibition under adherent culture conditions was <15% for most NSCLC cell lines and <10% for melanoma cell lines treated with 100 μM Lunasin, whereas inhibition of colony formation by human NSCLC and melanoma cell lines treated with 100 μM Lunasin ranged from ^~^65% to 85%, and ^~^20 to 40%, respectively. These results demonstrate that the Lunasin sensitivity of human and mouse lung cancer and melanoma cells are quite similar *in vitro*.

### *In vivo* effects of Lunasin treatment

**Figure 2.**
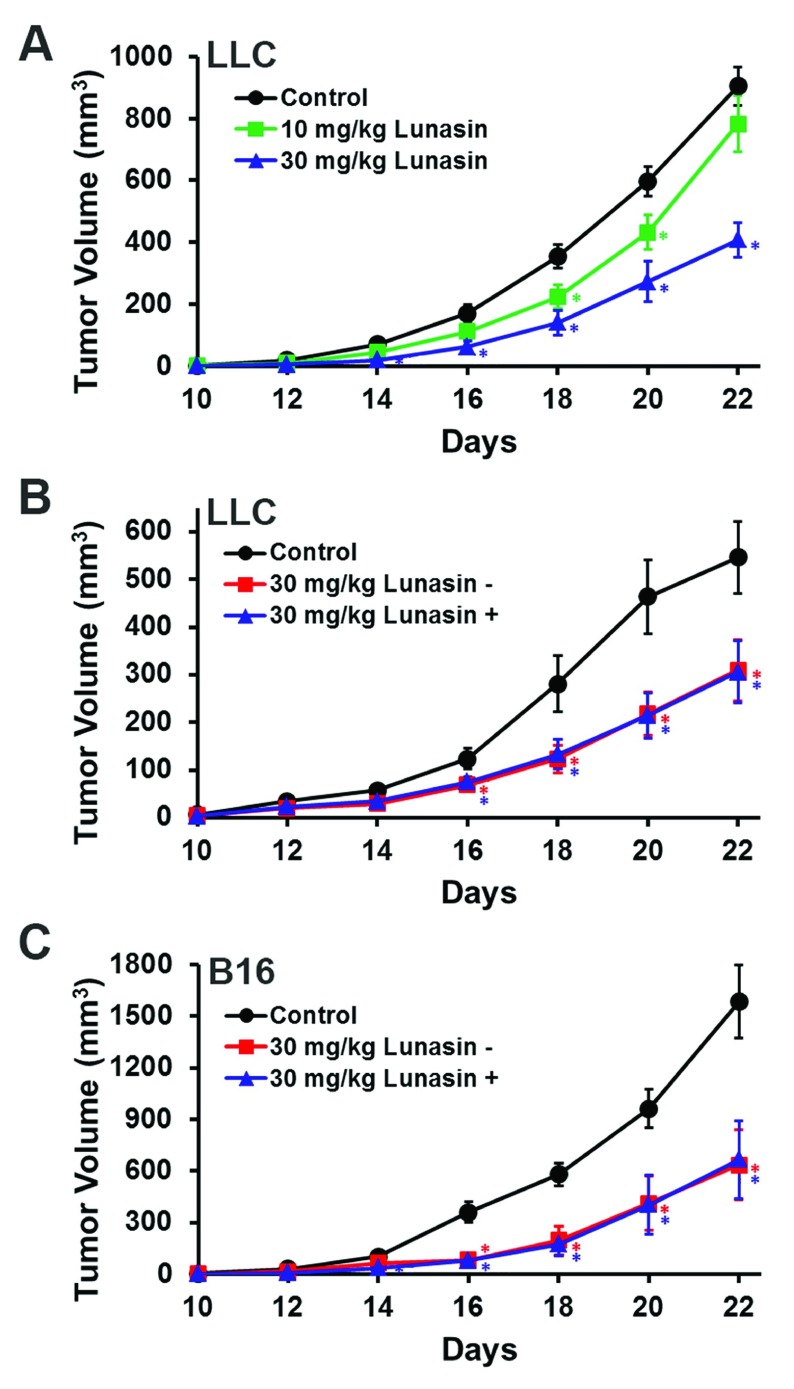
Lunasin inhibition of LLC and B16-F0 tumor growth in C57BL/6 mice. ( **A**) Effects of 10 mg/kg and 30 mg/kg Lunasin treatment on LLC tumor growth. ( **B**) Effects of 30 mg/kg Lunasin on the growth of tumors initiated by LLC cells either not pre-treated (Lunasin-, red) or pre-treated with 100 μM Lunasin (Lunasin+, blue) for 72 hours prior to injection of cells into mice. ( **C**) Effects of 30 mg/kg Lunasin on the growth of tumors initiated by B16-F0 cells either not pre-treated (Lunasin-, red) or pre-treated with 100 μM Lunasin (Lunasin+, blue) for 72 hours prior to injection of cells into mice. LLC (1 × 10 ^5^) or B16-F0 (1 × 10 ^6^) cells were injected subcutaneously in the hind flanks of mice to initiate tumors. Lunasin treatments were initiated on the same day that cells were injected and continued daily until the end of the experiment. Tumor volumes were determined from caliper measurements. Treatment groups contained 6–10 mice per group. The data shown represent the mean ± SEM and an asterisk (*) indicates that an individual treatment was significantly different ( *p* < 0.05) from the control as determined by an unpaired student’s *t-*test.

We initially tested the ability of Lunasin to inhibit tumor growth initiated by LLC cells at doses of 10 and 30 mg/kg. These doses are comparable to those for several biologic drugs and the cyclic peptide, cilengitide ^27, 28^. Lunasin inhibited tumor growth in mice treated at the 30 mg/kg dose by 55% at day 22 whereas the 10 mg/kg dose had only modest effects that were only statistically significant on days 18 and 20 ( Figure 2A, Table 3). We next tested whether pre-treating LLC cells with 100 μM for 72 h *in vitro* prior to implantation further affected tumor growth. This was prompted by our earlier studies demonstrating that Lunasin reduces the putative cancer initiating cell pool in human melanoma cell lines ^8^. The results clearly show that pre-treatment did not enhance inhibition of tumor growth by Lunasin at a dose of 30 mg/kg ( Figure 2B, Table 4). In this experiment, tumor growth at day 22 was 43% of the control. The inhibition of LLC tumor growth by Lunasin was somewhat less than that observed in xenograft studies of NSCLC H1299 where tumor growth was reduced by 63% at 32 days in mice treated with 30 mg/kg Lunasin ^15^.

Lunasin at a dose of 30 mg/kg was also found to inhibit tumor growth initiated by B16-F0 melanoma cells, with a reduction in tumor growth of 60% at day 22 ( Figure 2C, Table 5). As was the case with LLC, pre-treatment with 100 μM Lunasin for 72 h *in vitro* did not enhance inhibition of tumor growth. These results are quite comparable to our xenograft studies using the human melanoma cell line A375 where we observed a 55% reduction in tumor volume 34 days after implantation ^8^.

Dataset 1. Raw data of validation of syngeneic mouse models of melanoma and non-small cell lung cancer for investigating the anticancer effects of the soy-derived peptide lunasin All raw data are available in Table 1–Table 5. Click here for additional data file. Copyright: © 2017 Devapatla B et al. 2017 Data associated with the article are available under the terms of the Creative Commons Zero "No rights reserved" data waiver (CC0 1.0 Public domain dedication).

## Conclusion

These studies establish that syngeneic mouse models for lung cancer and melanoma are sensitive to Lunasin and that their sensitivity is comparable to that observed in xenograft studies of human NSCLC and melanoma. Thus, these models may be useful to further elucidate the mechanisms of Lunasin action, particularly potential immune effects, and provide important new information on the feasibility of using Lunasin to treat these two deadly cancers.

## Data availability

The data referenced by this article are under copyright with the following copyright statement: Copyright: © 2017 Devapatla B et al.

Data associated with the article are available under the terms of the Creative Commons Zero "No rights reserved" data waiver (CC0 1.0 Public domain dedication). 

F1000Research: Dataset 1. Raw data of validation of syngeneic mouse models of melanoma and non-small cell lung cancer for investigating the anticancer effects of the soy-derived peptide lunasin, 10.5256/f1000research.9661.d136965 ^29^
