# Supplementary material for: Validation of syngeneic mouse models of melanoma and non-small cell lung cancer for investigating the anticancer effects of the soy-derived peptide Lunasin
Source: F1000Res. 2017 Feb 22;5:2432. Originally published 2016 Oct 4. [Version 2] doi: 10.12688/f1000research.9661.2 (PMC5325107; doi:10.12688/f1000research.9661.2)
Supplement: Peer review history

# Peer review: Referee response for version 2

**DOI:** 10.5256/f1000research.11815.r20445

**Published:** 2017-02-22

**Authors:** Elizabeth S. Yeh

**Affiliations:** 1 Department of Cell and Molecular Pharmacology and Experimental Therapeutics, Medical University of South Carolina, Charleston, SC, USA

The authors have addressed the concerns raised in the original review.

I have read this submission. I believe that I have an appropriate level of expertise to confirm that it is of an acceptable scientific standard.

# Peer review: Referee response for version 1

**DOI:** 10.5256/f1000research.10410.r18085

**Published:** 2016-11-30

**Authors:** Elizabeth S. Yeh

**Affiliations:** 1 Department of Cell and Molecular Pharmacology and Experimental Therapeutics, Medical University of South Carolina, Charleston, SC, USA

The manuscript would be improved by addressing the following suggestions:

1. Is it known how Lunasin is penetrating the cell membrane or whether it is acting on an extracellular receptor?
2. With in vivo studies using peptides, it is difficult to assess bioavailability due to lack of stability. The manuscript would benefit from discussing what is known about in vivo delivery. This may take into account why pre-treatment of cells was no more effective than s.c. delivery.

I have read this submission. I believe that I have an appropriate level of expertise to confirm that it is of an acceptable scientific standard, however I have significant reservations, as outlined above.

# Response

**Published:** 2017-02-13

**Authors:** Keith Davis

**Affiliations:** The Johnson Center for Entrepreneurship in Biotechnology, Indiana University, USA

We thank Dr. Yeh for her review and comments.

 1. Is it known how Lunasin is penetrating the cell membrane or whether it is acting on an extracellular receptor?

*We do not currently know all the precise mechanisms whereby Lunasin may enter the cell. There are data that strongly support the hypothesis that lunasin can enter cells via the recycling of integrin receptors; however, how Lunasin is released internally and makes it way to the nucleus is not known.*

*Our studies suggest that it may not be necessary for Lunasin to enter the cell to exert at least some of its effects given that the inhibition of integrin signaling likely occurs at the cells surface.*

*We have added additional details in the Introduction to address this question.*

 2. With in vivo studies using peptides, it is difficult to assess bioavailability due to lack of stability. The manuscript would benefit from discussing what is known about in vivo delivery. This may take into account why pre-treatment of cells was no more effective than s.c. delivery.

*There is very little information available on the pharmacokinetics and bioavailability of Lunasin in vitro or in vivo, particularly with regard to the purified Lunasin peptide.  Human subjects fed 50 g of soy protein per day were found to have detectable levels of Lunasin in their blood.  Based on the amount of Lunasin thought to be present in the soy protein and estimates of loss due to digestion, the authors concluded that the average absorption rate of Lunasin in these subjects averaged 4.5%* *[1]* *. Another study suggested that Lunasin was also orally bioavailable in mice and that as much as 30% of the Lunasin reached the tested tissues intact* *[2]* *.*

*This is clearly an important area that needs significantly more work as we continue assessing the potential of Lunasin as an anticancer agent.*

*We have added the details of the available data on bioavailability in the Introduction.*

1. Dia, V.P., et al., *Presence of lunasin in plasma of men after soy protein consumption.* J Agric Food Chem, 2009. **57**(4): p. 1260-6.
2. Hsieh, C.C., et al., *Complementary roles in cancer prevention: protease inhibitor makes the cancer preventive peptide lunasin bioavailable.* PLoS One, 2010. **5**(1): p. e8890.

# Peer review: Referee response for version 1

**DOI:** 10.5256/f1000research.10410.r17556

**Published:** 2016-11-23

**Authors:** Masaki Terabe

**Affiliations:** 1 Vaccine Branch, National Cancer Institute (NCI), National Institutes of Health (NIH), Bethesda, MD, USA

Major points:

1. Are the concentrations of Lunasin used physiologically relevant?
2. Does Lunasin have similar effects on the progression of these tumors in immunodeficient mice such as RAG deficient mice and RAG/common gamma chain deficient mice? Comparing the effect of Lunasin in these two models, immunodeficient mice and immunocompetent mice, will provide information as to whether adaptive and innate immune cells are involved. Since there is a direct toxicity of Lunasin to tumor cells, it would be important to show the contribution of immune cells to the anti-tumor effect of Lunasin seen in the in vivo models.

Minor point:

1. Axis labels of Fig 1 may not be correct. Are those really % control?

I have read this submission. I believe that I have an appropriate level of expertise to confirm that it is of an acceptable scientific standard, however I have significant reservations, as outlined above.

# Response

**Published:** 2017-02-13

**Authors:** Keith Davis

**Affiliations:** The Johnson Center for Entrepreneurship in Biotechnology, Indiana University, USA

We thank Dr. Terabe for his review and comments.

 1. Are the concentrations of Lunasin used physiologically relevant?

*Our view is that it is difficult to assess the physiological relevance of potential therapeutics in the absence of detailed pharmacokinetic data, particularly when assessing in vitro assays, which often are not very predictive of in vivo effects.  We assume that Lunasin is not particularly stable in cell cultures where a number of proteases are present, thus; it is not surprising that concentrations of 100 µM are required for significant activity.  With respect to the in vivo studies, a dose of 30 mg/kg body weight is similar to that of several biologic drugs, as well as the RGD-peptide drug cilengitide* *[1]* *. Given our initial preclinical data in mice showing that daily injection of 30 mg/kg Lunasin does not induce any signs of toxicity while providing a therapeutic effect* *[2]* *suggest that the dosage of Lunasin used in vivo is potentially physiologically relevant. Clearly, more studies are needed to validate this conclusion in humans, and it is very likely that substantially more work will need to be done to find appropriate formulations and/or modifications of the Lunasin peptide before it would have potential clinical utility.*

 2. Does Lunasin have similar effects on the progression of these tumors in immunodeficient mice such as RAG deficient mice and RAG/common gamma chain deficient mice? Comparing the effect of Lunasin in these two models, immunodeficient mice and immunocompetent mice, will provide information as to whether adaptive and innate immune cells are involved. Since there is a direct toxicity of Lunasin to tumor cells, it would be important to show the contribution of immune cells to the anti-tumor effect of Lunasin seen in the in vivo models.

*We have not done these studies, nor are we aware that they have been done by others.  We agree that using mouse genotypes with various levels of immunodeficiency would be an excellent way to address the potential immune modulatory effects of Lunasin. However, this is beyond the scope of this research note.*

  Minor point:

 1. Axis labels of Fig 1 may not be correct. Are those really % control?

*The original graph did use a decimal representation of the data rather than the percentage.  We have modified the axis to represent the percentage values to be consistent with the axis label.*

  

1. Nabors, L.B., et al., *Two cilengitide regimens in combination with standard treatment for patients with newly diagnosed glioblastoma and unmethylated MGMT gene promoter: results of the open-label, controlled, randomized phase II CORE study.* Neuro-Oncology, 2015. **17**(5): p. 708-717.
2. Shidal, C., et al., *Lunasin is a novel therapeutic agent for targeting melanoma cancer stem cells.* Oncotarget, 2016 7(51): p. 84128-84141.

# Peer review: Referee response for version 1

**DOI:** 10.5256/f1000research.10410.r17429

**Published:** 2016-11-07

**Authors:** Jean-Pierre Gillet

**Affiliations:** 1 Laboratory of Molecular Cancer Biology, Molecular Physiology Research Unit-URPhyM, Namur Research Institute for Life Sciences (NARILIS), Faculty of Medicine, University of Namur, Namur, Belgium

This manuscript would benefit from further discussion on:

1. The clinical relevance of the Lunasin concentration used in the mice studies.
2. The rationale and the relevance of pretreating the cell lines with Lunasin prior to mouse engraftment.
3. The pros/cons of this model compared with GEMM (including regulatory agency recommendations to better appraise the current drug development environment).

I have read this submission. I believe that I have an appropriate level of expertise to confirm that it is of an acceptable scientific standard, however I have significant reservations, as outlined above.

# Response

**Published:** 2017-02-13

**Authors:** Keith Davis

**Affiliations:** The Johnson Center for Entrepreneurship in Biotechnology, Indiana University, USA

We thank Dr. Gillet for his review and suggestions.

 1. The clinical relevance of the Lunasin concentration used in the mice studies.

*As discussed in our response to Dr. Terabe’s comments, it is difficult to assess the clinical relevance of the 30 mg/kg body weight dose used in our studies beyond the fact that this dose is comparable to that used for other protein and/or peptide drugs and that it is not unusually high when compared to a number of other agents being tested at the preclinical stage.*

 2.  The rationale and the relevance of pretreating the cell lines with Lunasin prior to mouse engraftment.

*The rationale for pretreating the cells prior to engraftment was based on our recent studies demonstrating the ability of Lunasin to reduce the putative cancer initiating cell population of human melanoma cell lines (as identified as being ALDH ^high^)* *[1]* *.  We have subsequently not pursued this avenue of research with the B16-F10 or LLC cells so we cannot provide any further data on whether any ALDH ^high^ cells that may be present in these cells lines were affected by Lunasin.*

*We have added the rational for the pretreatment to the Results and Discussion.*

 3.  The pros/cons of this model compared with GEMM (including regulatory agency recommendations to better appraise the current drug development environment).

*Dr. Gillet raises a good point, given that the landscape of preclinical cancer research has changed significantly with the development of transgenic mouse models for a variety of cancer types, and in some cases, subtypes of specific cancers. It is well known that traditional xenograft studies using established human cell lines are often not predictive of clinical efficacy, which can also be the case for the B16 and LLC syngeneic models. However, these more traditional models do still have a role in early preclinical studies when one requires a rapid and relatively inexpensive method to obtain an initial assessment of a compound’s anticancer activity in vivo* *[2, 3]* *. In the case of xenograft studies, there has been a resurgence of use as they represent a convenient system for maintaining and studying patient-derived tumors.  Our decision to test the syngeneic B16 and LLC models was based primarily on identifying a model where we could use a fully immunocompetent mouse without establishing the rather costly GEMM and humanized mouse models.  Indeed, now that we have significant results in human xenograft and mouse syngeneic models, we hope to be able to extend our studies into appropriate GEMM models as the next logical step leading towards clinical testing.*

*We have not significantly modified the manuscript to address this point, other than to modify the Conclusion to limit the likely utility of using these syngeneic models.  The purpose of this research note is simply to inform researchers interested in Lunasin that the B16 and LLC models do respond and may be appropriate for their studies, depending on the goal. We feel that a more detailed discussion of the pros and cons and these models compared to GEMM and humanized mice models would be more appropriate for a full research paper*.  

  

1. Shidal, C., et al., *Lunasin is a novel therapeutic agent for targeting melanoma cancer stem cells.* Oncotarget, 2016 7(51): p. 84128-84141.
2. Richmond, A. and Y. Su, *Mouse xenograft models vs GEM models for human cancer therapeutics.* Dis Model Mech, 2008. **1**(2-3): p. 78-82.
3. Talmadge, J.E., et al., *Murine models to evaluate novel and conventional therapeutic strategies for cancer.* Am J Pathol, 2007. **170**(3): p. 793-804.
